# Neurobiological emergentism: sentience as an emergent process and the experiential gap

**DOI:** 10.3389/fpsyg.2025.1528982

**Published:** 2025-08-01

**Authors:** Todd E. Feinberg

**Affiliations:** Department of Psychiatry, Icahn School of Medicine at Mount Sinai, New York, NY, United States

**Keywords:** neurobiological emergentism, biological emergence, animal sentience, consciousness, evolution of sentience, explanatory gap, experiential gap

## Abstract

One of the most controversial and debated problems regarding the nature of sentience, is how to integrate the biology and neurobiology of sentience with the problem of the “explanatory gaps” that are proposed to arise between the functions of the nervous system as *objectively and scientifically explained* and sentience—or more generally consciousness—as it is *subjectively experienced*. In this paper I discuss a theory I have called *Neurobiological emergentism (NBE)* that is based upon a *biological-neurobiological-evolutionary* model that explains both how sentience *emerges* from complex nervous systems as well as scientifically resolves the explanatory gaps. I propose a model in which the emergence of sentience occurs roughly in three stages: Emergent Stage 1 (ES1) single-celled *sensing* organisms without neurons or nervous systems that appeared approximately 3.5–3.4 billion years ago and are *non-sentient*; Emergent Stage 2 (ES2) *presentient* animals that appeared approximately 570 million years ago (mya) that have neurons and simple nervous systems and fall between ES1 and ES3 animals; and Emergent stage 3 (ES3) *sentient* animals that emerged along diverse evolutionary lines during the Cambrian period approximately 560–520 mya, a group that includes all vertebrates (fish, reptiles, birds, and mammals), arthropods (insects and crabs), onychophorans (velvet worms) and cephalopods such as the octopus and squid that possess neurobiologically complex central nervous systems. I describe how this model leads to a scientific resolution of two related “explanatory gaps” (*the personal nature of sentience* and the *character of experience*), both of which are created by the natural emergence of sentience. However, in place of the “explanatory gaps,” I propose that there is an *experiential gap* that emerges between the objective brain and subjective experience, but that this “gap” can be fully scientifically explained and naturalized and can account for the personal subjective nature of sentience without completely “objectifying” it.

## 1 Introduction: what is sentience?

There are various terms and definitions in the scientific and philosophical literature that have been used to describe “consciousness.” In this paper, I review my theory of *Neurobiological emergentism* (*NBE*) (Feinberg, 2024) that is aimed specifically at providing a scientific explanation of the *subjective, feeling* aspects of consciousness, what the philosopher Nagel (1974) called “something it is like to be.” While these more experiential aspects of “consciousness” have been referred to by various names such as *primary consciousness, phenomenal consciousness*, or *sensory consciousness* (Feinberg and Mallatt, 2016, 2018, 2020; Feinberg, 2024; Revonsuo, 2006, 2010) here I will refer to these states as simply *sentient.*

Various experiential features have been proposed to define the nature of sentience across species. One of the most widely used ways of qualifying sentient experiences is that they possess *valence* that is characterized by an inherent *positivity* (pleasantness) or *negativity (*unpleasantness*)* of a sensory experience (Adolphs and Anderson, 2018 see below). However, I think a view that is more consistent with the meaning of the term *sentience*, as defined by the presence of “feeling,” would include *all* types of subjective felt experiences. By this criterion, sentience would encompass not only *interoceptive-affective* feelings (pain, pleasure, and emotions etc.) that are characterized by feelings that are internally generated and possess inherent “valence,” but also *exteroceptive sensory experiences* (vision, audition, olfaction, taste etc.) that represent externally generated feelings from the world that do not necessarily carry emotional “valence” but are nonetheless *feeling states* that entail “something it is like to be.”

I believe that the sentient aspects of consciousness are the most refractory to scientific explanation as well as being largely responsible for the scientifically and philosophically refractory problem of what has been referred to as the “explanatory gap” between the *objective* scientific facts of biology and the *subjective* personal nature of feeling (Chalmers, 1995, 2018; Feinberg and Mallatt, 2016, 2018, 2020; Feinberg, 2024; Revonsuo, 2006, 2010; Levine, 1983; Ludlow et al., 2004; Searle, 1992, 1997).

I have proposed a theory called *Neurobiological emergentism (NBE)* (Feinberg, 2024) that is based upon a *biological-neurobiological-evolutionary* model of the emergence of sentience. I argue that by viewing sentience as a biologically emergent property of sufficiently complex brains we can explain both the biological basis of sentience as well as its subjective nature. I begin in the next section to describe what aspects of emergence I focus on.

## 2 Emergence in biological systems

The first use of the term emergence in the scientific literature is most commonly credited to G.H. Lewes from his book *Problems of Life and Mind* that was published (Lewes, 1875; Clayton, 2006). The presence of emergence emphasizes how features of a system may arise from or “emerge” as a result of the functions of the parts of a system and their interactions. Especially important for explaining the unique aspects of sentience is that since an emergent feature is a “higher-level” property of a complex system that is created by the collective functions of that system’s parts, it follows that it is a *novel system feature* that is not entirely reducible to the individual parts that create it. This is why it is commonly stated that an emergent feature is more than the “simple sum” of the features of its individual or “lower level” parts (Ahl and Allen, 1996; Allen and Starr, 1982; Beckermann et al., 2011; Bedau, 1997; Bedau and Humphreys, 2008; Clayton and Davies, 2006; Feinberg and Mallatt, 2020; Feinberg, 2024; Gibb et al., 2019; Morowitz, 2002; Nunez, 2016; Pattee, 1970; Salthe, 1985; Searle, 1992, 1997; Vintiadis, 2013; Feinberg, 2012).

Emergent features in biological systems are universal. Indeed “life” itself and the processes of “living” are *emergent biological properties* of inanimate matter. And since my primary interest here is to explain the emergence of sentience in living organisms, NBE focuses on the emergent biological properties that are commonly found in all living things. The main features of biological emergence are summarized in Table 1.

**TABLE 1:** Major general features of biological emergence.

• An emergent feature is an *aggregate system feature* that is not present in the parts. Thus, emergent properties are *novel* in comparison to the properties of the parts that create them
• In order for emergence to occur as an aggregate system feature, the individual parts of the system must be *physically united, integrated or at a minimum interacting in some fashion*
• Emergent features are *processes* created by the dynamic interaction of the system’s parts
• *Hierarchical systems* are critical to and increase emergent properties

Adapted from Feinberg and Mallatt (2020), licensed under CC BY 4.0.

One of the postulates of NBE is that the *neurohierarchical connections and their processes* are absolutely essential for the creation and emergence of sentience. This is because, in order for emergence to occur as an aggregate and novel system feature, the individual parts of the system must be extensively interacting directly or indirectly in some manner. Therefore, it follows that the emergence of novel features is potentially greatly magnified in neurobiological hierarchies in which many “lower system levels” influence “higher system levels,” which in turn influence the lower levels, and structures within the same level also influence each other via extensive reciprocal connectivity. The increase in the magnitude of these interactions in more evolved and complex brains will prove to be essential for the emergence of sentience. The key features of emergence in neurobiological hierarchical systems are listed in Table 2.

**TABLE 2:** Major features of emergence in neurobiological hierarchical systems.

• *Hierarchical arrangements* are important for the creation of emergent features in all of biology. This is especially true of neurohierarchical systems
• *Reciprocal connections* among parts within and between levels of biological hierarchies greatly enhance the emergence of novel properties in biological and neurobiological hierarchies
• Biological emergent properties may occur *simultaneously* at multiple spatial and scalar levels and across diverse temporal frequencies
• *Novel properties* emerge in a *system as a whole* as additional (typically “higher”) levels are added

Reproduced from Feinberg (2024), licensed under CC BY-NC-ND 4.0.

## 3 The emergence of sentience and the “explanatory gaps”

In later sections I will elaborate in greater detail the specifics of the NBE model of the emergence of sentience. But first I discuss what philosophical “gaps” I believe NBE can help explain. In my view the relationship between the science of sentience and the philosophical issues it raises can be broken down into two related, partially overlapping but somewhat different, “explanatory gaps,” both of which I argue biological emergence can scientifically explain.

### 3.1 The first gap: the “personal nature” of sentience

First, one of the most important elements that contributes to the “mysterious” nature of sentience is how to integrate the *objectively* described biology and neurobiology of sentience with its *subjective* personal nature. This is one of the most fundamental aspects of the problem of explaining the relationship between the nervous system and sentience.

Philosopher C. D. Broad (1887–1971) provided one of the earliest and most influential illustrations of the apparent gap between the physical brain as objectively observed versus how it is subjectively experienced. Nearly 100 years ago, in his book *The Mind and Its place in Nature* (Broad, 1925), Broad presented what would become a now famous thought experiment that illustrates the personal nature of sentience and the “mind-body” problem.

Broad’s premise involves the personal experience of smelling ammonia. He proposed that even if an omniscient “mathematical archangel” had total “objective” knowledge of the chemistry of ammonia and also had full knowledge of the neurobiological basis of the smell pathways from olfactory nerve to the brain, the archangel still could not predict nor know what the subjective smell of ammonia would actually be like without *personally experiencing* and had smelled it for himself.

Much later on, philosopher John Searle referred to the personal nature of consciousness as the *first-person* or *subjective ontology of consciousness.* His argument was that any state of consciousness is irreducibly and ontologically someone’s or some animal’s first-person state and that objective brain functions and subjective experience are mutually irreducible because in each case you would leave out either the objectivity or subjectivity that is in question (Searle, 1997).

However, while Searle’s view was that there were *irreducible* differences between the nervous system and subjective experience, he did not feel that this apparent divide or “gap” between objective knowledge of the brain and the first-person experience posed a problem for a fully scientific and objective explanation of consciousness. However, it remained scientifically unexplained how this gap is created in the first place. I refer to this first philosophical gap as the problem of the *personal nature of sentience.*

### 3.2 The second gap: the “subjective character” of experience

A second “gap” that is related to the first is how can we explain what is commonly referred to as the subjective *character of experience*. One of the best-known analyses of this problem was presented by philosopher Levine in his 1983 paper entitled *Materialism and qualia: The explanatory gap.* It was in this paper that he first introduced the term the “explanatory gap.”

Along similar lines to Broad’s archangel and Searle’s ontological subjectivity, Levine also noted that there appeared to be an explanatory gap between the neural processes of the *physical brain* and the subjective *experiences* associated with these brain processes. But this is a somewhat different aspect of the personal nature of sentience that relates more directly to the problem of the *subjective character of experience:* Why do brain states “feel” the particular way that they do? In his example, the connection between “C-fiber firing” and the feeling of pain seems “completely mysterious’ (Levine, 1983). More recently, philosopher Chalmers (1996) opined that Levine’s “explanatory gap” was central to what he called the “hard problem” of consciousness that entails aspects of *both* Broad’s and Levine’s concerns.

In summary, I think that what has been generally known as the “explanatory gap,” or what Chalmers called more broadly the “hard problem” of consciousness, is actually two related problems. The first, as described by Broad and Searle, is the *personal nature of sentience*, and this is the apparent gap between the *objective description* of the biological basis of an experience and actually *having* that experience. The second is the *character of experience:* why and how certain brain states create the specific feelings that they do. So how can these two problems be resolved and explained within a single theory that does not entail any novel scientific laws or principles? And how might they relate to the principles and tenets of emergence theory in biological systems that I discussed above?

### 3.3 “Strong” versus “weak” emergence and the “explanatory gap”

The challenges of relating the complex neurobiological emergence of sentience with *both* its personal nature and the unique character of “feelings” have led some philosophers of consciousness to argue that no standard version of biological emergence—such as NBE—could ever explain the biologically natural emergence of sentience.

For instance, one of these more extreme views on this issue has been called “strong” or “radical” emergence. This is the assertion that sentience and consciousness must be the result of some novel “fundamental” *physical property or process*. For instance, Chalmers (1995) has proposed one version of a strong emergence theory of consciousness and proposed that there must be some *fundamental novel physics* at work in the creation of consciousness and hence sentience. While he does not describe what this “fundamental feature of the world, alongside mass, charge, and space-time” is that makes consciousness possible, as far as what “type” of emergence is involved, he expresses the view that the “phenomenon of consciousness” is a unique instance of “strong emergence” (Chalmers, 2006).

In my view, there is little doubt that this type of “strongly emergent” hypothesis would inevitably lead to a variety of *mind-body dualism* in which it is argued that mind—in contrast to the body or brain—is in some sense “non-physical.”

And indeed, Chalmers arrived at a philosophical position he called *naturalistic dualism* (Chalmers, 2017). In this theory, he proposed that sentient states “naturally supervene” (depend upon) physical systems such as brains; but at the same time, he asserted that mental states are *ontologically distinct* and not “reducible” to physical systems.

In contrast to a “strong” emergence view of sentience or consciousness, NBE is a “weak” emergence theory, meaning that standard (“weak”) biological emergent processes are all that are required to explain the natural and wholly physical emergence of sentience. Thus, NBE offers a version of emergence of sentience that is not different “in kind” from the principles of emergence in biological (Table 1) or neurobiological (Table 2) systems in general.

A similar, earlier hypothesis sheds additional support for a “natural” role of emergence in complex hierarchical systems in the creation of novel system properties. Contextual Emergence Theory describes how emergence occurs within a complex system via the reciprocal relationships between its “higher” and “lower” levels (Atmanspacher and beim Graben, 2009; Atmanspacher, 2015; beim Graben, 2014; Bishop and Atmanspacher, 2006). One of its major postulates is that *reductionist* arguments about the properties of a non-linear physical system fail to adequately consider the emergent properties of that system because the system’s higher-level conditions—what the authors call the “contingent context”—reciprocally influence the system’s lower-level mechanics, so the “lower levels” alone cannot explain the emergent properties of the whole. This is another reason the emergence of sentience requires complex hierarchical nervous systems.

## 4 Varieties of sentience

As I mentioned in the Introduction, I believe that there are varieties of sentience, and that these can be roughly divided into two subtypes: feelings that are generated from the world (*exteroceptive sentience*) and those that are more internally generated (*interoceptive-affective sentience*; pain and pleasure, emotions, etc.) (Feinberg and Mallatt, 2016, 2018). There is both commonality and diversity among these different subtypes of emergent feelings that I discuss below (section 5).

### 4.1 Exteroceptive sentience

**FIGURE 1:**
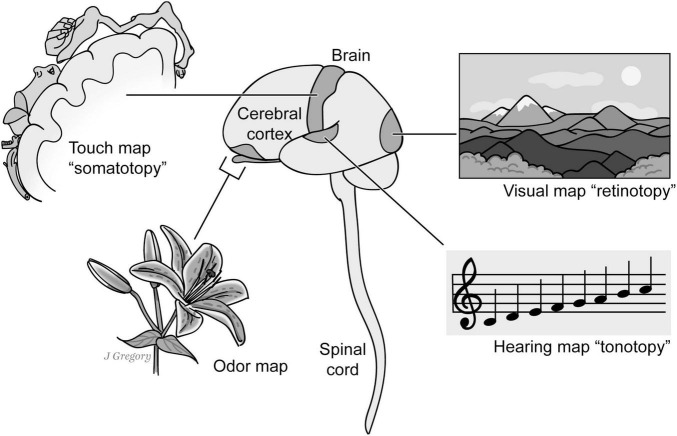
Exteroceptive sentience, topographical maps and the creation “mental images.” One criterion for the presence of exteroceptive sentience is that an organism or animal is capable of not only basic sensing capabilities such as mechanical sensation such as touch and proprioception, chemosensation including taste and smell, vision, hearing, etc. but it is also able to create *sensory mental images* based upon the brain’s capacity to create unified higher order *mapped neural representations.* Reproduced from Feinberg (2024), licensed under CC BY-NC-ND 4.0.

Exteroceptive sentience is defined as the capacity of an organism to possess not only basic *sensing* capabilities (for example the capacity to sense a feature of the environment via vision (photosensation), mechanical sensation (touch and proprioception), chemosensation (including taste and smell), etc.) but also exteroceptive *sentient* “feelings” associated with these sensing processes that are a critical part of sentience. From this it follows that the creation of “sensory mental images” that go beyond basic and reflexive sensing functions serve as a good marker for the presence of *exteroceptive sentience* (Edelman, 1992; Damasio, 2000, 2010; Feinberg and Mallatt, 2016, 2018; Figure 1).

The primary neurobiological evidence for the presence of an exteroceptive mental image is that in order for a nervous system and a brain to create a unified sensory mental image, there must be some higher order *mapped neural representations* in a particular sensory domain (Figure 1). For example, classic examples of a mapped neural representation are called *topographical* maps in which a spatial ordering is hierarchically preserved from the lowest level of the sensory field to the higher levels in the central nervous system. One classic example is the *somatotopic* map for the touch-related senses that roughly preserves the spatial location of a stimulus on the body; another is the *retinotopic* map where the visual field is initially spatially mapped in the retina and likewise “retinotopically” in the brain. However, not all sensory “brain maps” reflect the locations of stimuli in physical space. For instance, the auditory *tonotopic* map is spatially organized according to sound frequencies and the chemosensory maps senses of taste and smell are primarily organized around chemical receptor properties

### 4.2 Interoceptive-affective sentience

A second type of sentience is the presence of *interoceptive* and *affective* feelings. In contrast to exteroceptive sentience that entails the processing of information from the external environment, *interoceptive* feelings include sensations such as pain, hunger, thirst, motivational drives, etc. and are thus more responsive to physiological changes in the body.

Finally, *affective* awareness is a broad category that covers all types of emotional experiences. In contrast to the tight mapped neural representations that characterize exteroceptive awareness, interoceptive-affective experiences do not require this sort of succinct neural mapping, and their underlying neurobiologies involve different neuroanatomical structures that are also more widely distributed when compared to exteroceptive mappings.

Another significant difference between exteroceptive versus interceptive-affective feelings, as noted above, is that while exteroceptive sentience does not in itself typically carry positive or negative feelings to its sensory images, interoceptive-affective sentience does. So sentient interoceptive-affective experiences are said to have intrinsic *valence* (Berridge, 2018, 2019; Berridge and Kringelbach, 2015; Cabanac, 1996; Cabanac et al., 2009; Damasio, 2000, 2010; Denton, 2006) that Adolphs and Anderson (2018, p. 66) defined as a critical feature of emotional experience that represents a *psychological dimension* of the “pleasantness or unpleasant” or the response features of an “appetitive or aversive” response to a stimulus.

In summary, distinguishing these diverse subtypes of “feelings” is important for a complete account of sentience. Additionally, the emergentist approach of NBE can explain why there is no single underlying neuroanatomical pathway type—whether across species or even within a single animal (e.g., exteroceptive versus interoceptive-affective)—that is required for the creation of sentience because these various sentient experiences *all have in common* that they are the products of diverse emergent processes in neurobiologically complex brains. I discuss this point in greater depth in the next section.

## 5 A three-stage model of the biological-neurobiological-evolutionary emergence of sentience

**FIGURE 2:**
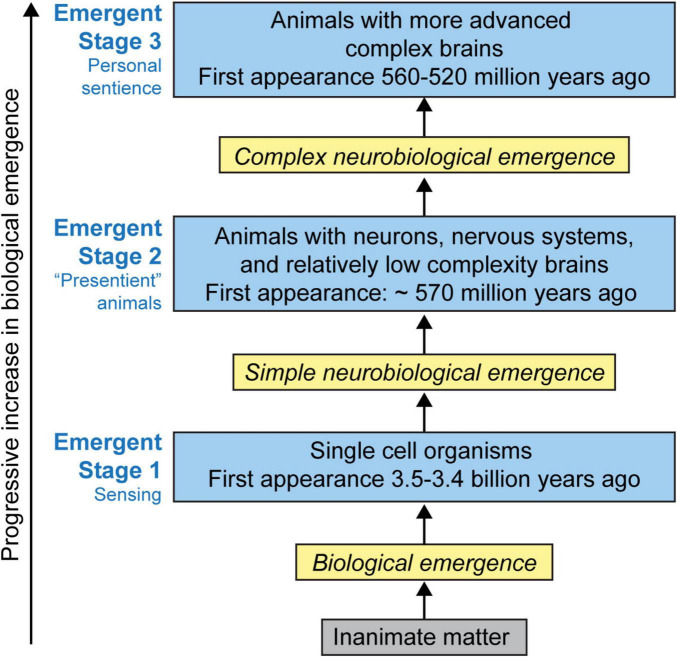
The stages in the emergence of sentience. It is proposed that there are roughly three stages: Emergent Stage 1 (ES1); Emergent Stage 2 (ES2); and Emergent Stage 3 (ES3) in the emergence of sentience. Sentience is hypothesized as a naturally occurring emergent feature of life, and that emergence progresses from sensing to sentience in stages as a result of increasing numbers of neurons, their specialized neural functions, and increasing *number* of neurohierarchical levels and their interactions (Table 4). It is hypothesized that the *personal nature of sentience* is a natural result of this progression. Reproduced from Feinberg (2024), licensed under CC BY- NC-ND 4.0.

In order to trace the relationship between the biology, neurobiology, evolution, and philosophy of the explanatory gaps, I propose the progression of sensing to sentience can be roughly divided into three emergent stages: Emergent Stage 1 (ES1; *Sensing*); Emergent stage 2 (ES2; *Presentient*); and Emergent stage 3 (ES3; *Sentient*) with the progressive evolution of sentience occurring in punctuated transitions among these stages (Figure 2).

### 5.1 Emergent Stage 1 (ES1): non-sentient but sensing single-celled organisms

NBE proposes that the neurobiological mechanisms that create sentience are emergent features of the embodied life of the organism. If this view is correct, it follows that any theory of the emergence of sentience—at least all animal sentience—must begin with the emergence of life (Figure 2).

Some philosophers who are also interested in explaining consciousness have expressed similar views on the relationship between life and consciousness. Most notably, philosopher Thompson in his book *Mind in Life* argued that the relationship between life processes and consciousness is a significant factor in creating the aforementioned “explanatory gap”:

I have argued that the standard formulation of the hard problem is embedded in the Cartesian framework of the “mental” versus the “physical,” and that this framework should be given up in favor of an approach centered on the notion of life or living being. Although the explanatory gap does not go away when we adopt this approach, it does take on a different character. The guiding issue is no longer the contrived one of whether a subjectivist concept of consciousness can be derived from an objectivist concept of the body. Rather, the guiding issue is to understand the emergence of living subjectivity from living being, where living being is understood as already possessed of an interiority that escapes the objectivist picture of nature (Thompson, 2007, p. 236).

I believe Thompson’s philosophical point of view is consistent with and lends support for the view I have presented here: embodied life ultimately gives sentience its naturally evolving personal nature (Feinberg and Mallatt, 2020). But, as Thompson noted in the above quote, the problems posed by the explanatory gap are not resolved or eliminated simply because consciousness or sentience is a feature of embodied life. That requires further explanation.

**FIGURE 3:**
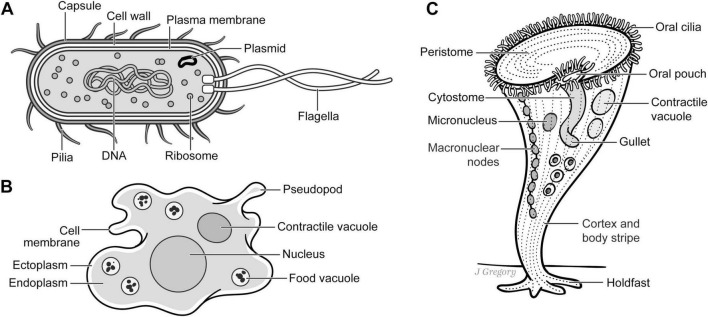
Emergent Stage 1 single-celled organisms. **(A)** *E. coli.* **(B)**. *Amoeba.* **(C)**. *Stentor roeseli*. Reproduced from Feinberg (2024), licensed under CC BY-NC-ND 4.0.

Hence, I propose Emergent Stage 1 (ES1) began the progression from life to sentience 3.5–3.4 billion years ago with the earliest living *single-celled organisms* that lacked neurons or nervous systems and evolved into modern-day prokaryotes such as *E. coli* and the eukaryotes *Amoeba* and *Stentor* Figure 3). These appeared as prokaryotes approximately 3.5–3.4 billion years ago and includes all single-celled organisms with *basic sensing capabilities* and responsiveness to the environment.

While I argue that these more basal ES1 organisms without neurons or nervous systems are not sentient, they clearly possess basic *sensing* capabilities. All living organisms, including plants and single-celled organisms, in order to survive, must have the capacity to adaptively respond to their external environment including responsiveness to such forces such as gravity and touch as well responsiveness to complex array of internal homeostatic features (Haswell et al., 2011). And these capabilities—in common with sentience—are emergent features of the animal’s embodied life processes.

**FIGURE 4:**
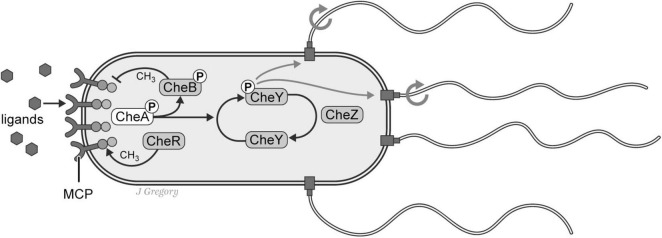
Chemotaxis pathways in the prokaryote *E. coli*. *E. coli* has complex sensing systems that enable it to adaptively respond to an array of environmental stimuli including the concentrations of nutrients and toxins. In this schematic illustration the organism responds to extracellular ligands that activate transmembrane chemoreceptors (MCPs) and response regulators (CheB, and CheY) in a complex coordination with phosphatase CheZ that determines the probability of “tumble or runs”. Reproduced from Feinberg (2024), licensed under CC BY-NC-ND 4.0.

Taking as an example, the ES1 single-celled bacterium *Escherichia coli* (*E. coli;* Figure 3A). Despite it possessing a relatively simple biological anatomy with no neurons, it is capable of a surprisingly complex array of adaptive behaviors. For instance, it can detect and adaptively respond to concentrations of nutrients and toxins, pH and oxygen levels, osmolarity, and the intensity and wavelength of light and temperature of its environment that is achieved via an array of biochemical signaling pathways (Kearns, 2010; Macnab, 1999; Micali and Endres, 2016; Patteson et al., 2015; Sagawa et al., 2014; Sourjik and Wingreen, 2012; Wadhams and Armitage, 2004; Figure 4).

**FIGURE 5:**
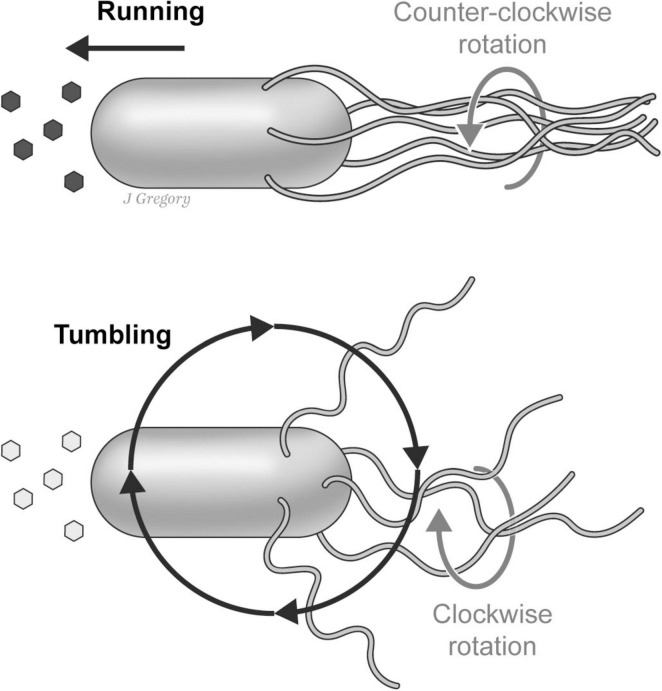
The “run-and-tumble” motion of an *E. coli*. In favorable environmental conditions the flagellar motors collectively rotate in a counterclockwise fashion causing the *E. coli* to “run” in a forward direction. When moving toward aversive conditions the motors switch to a clockwise direction causing the flagella to rotate in more independent directions that cause the *E. coli* to “tumble” thus altering its course. Reproduced from Feinberg (2024), licensed under CC BY-NC-ND 4.0.

The manner in which *E. coli* adaptively navigates in its watery environment is also remarkable. For its direction of locomotion, *E. coli* utilizes a system of 5–10 flagella that are randomly distributed on its cell surface. When environmental conditions are favorable, the flagellar motors rotate in a counterclockwise fashion that results in the bacterium swimming smoothly in a *forward* (“run”) direction. In contrast, when the organism is moving toward less favorable or adverse conditions, the motors switch to an opposite clockwise direction, causing the individual flagella to rotate in different and more independent directions resulting in a “tumble” that alters its course of direction (Egbert et al., 2010; Figure 5).

So, despite the lack of any neurons or nervous system, thanks to what is called this “run-and-tumble” motor system, *E. coli* is capable of adaptively responding to environmental conditions without actually “choosing” the direction of its motion. In other words, the *sensing* behaviors of *E. coli* are *reflexive* and are coordinated by hard-wired connections to the motor apparatus that results in the organism probabilistically ending up in more favorable conditions. Thus, while this sensorimotor behavior is adaptive, it is nonetheless entirely reflexive.

#### 5.1.1 ES1 organisms are sensing but not sentient

First, given the behaviors I discussed above, there is no question that single-celled organisms like *E. coli* are capable of *sensing.* However, they lack the neural architecture that is required for the creation of neurohierarchical sensory maps or their associated mental images. Second, while they are capable of adaptively responding to an assortment of positive and negative environmental stimuli, they do so without any of the neuroanatomical infrastructure that is required for interoceptive-affective feeling. Third, while the “sensing” behaviors of single-celled organisms are adaptive, they are nonetheless entirely reflexive, probabilistic, and are fully explained by the hard-wired molecular systems of the organism that are not different in kind from other adaptive biological processes like photosynthesis or other complex metabolic processes.

However, while I propose that single-celled organisms are sensing without sentience, these ES1 sensing organisms that emerged and evolved 3.5–3.4 billion years ago do set the stage for the eventual emergence of sentience in ES3 sentient animals 3 billion years later.

### 5.2 Emergent stage 2 (ES2): “Presentient” animals

**FIGURE 6:**
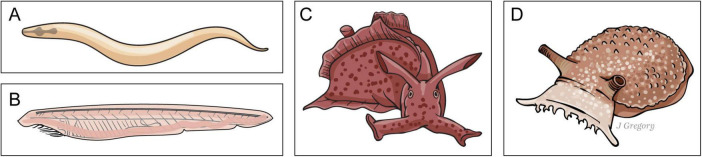
Emergent stage 2. Presentient animals. **(A)** *Caenorhabditis elegans.* **(B)** The cephalochordate *amphioxus.* **(C,D)**. *Aplysia and Pleurobranchaea* (sea slugs). (Illustration used with permission of ©Mount Sinai Health System).

The next emergent stage includes animals that made their first appearance approximately 570 million years ago. They have neurons and nervous systems, but these are far less complex, differentiated, and neurohierarchical nervous systems when compared to ES3 sentient animals. Representative ES2 species are the nematode worm *Caenorhabditis elegans;* the cephalochordate *amphioxus*, an animal on the phylogenetic line of sentient vertebrates; and the gastropod mollusks *Aplysia and Pleurobranchaea* (sea slugs) that are on the phylogenetic line leading to sentient coleoids such as octopus and squid (Figure 6).

I have described these ES2 animals as *presentient* and propose that they fall somewhere between basic sensing (ES1) organisms and sentient (ES3) animals along the evolutionary paths from sensing to sentience (Figure 2; Feinberg, 2024).

#### 5.2.1 The ES2 animal amphioxus: on the vertebrate phylogenetic line

Amphioxus (*Branchiostoma*) is a good representative of an ES2 animal. Amphioxus, also known as a “lancelet,” is a fish-shaped marine animal about 4–6 cm long. It is a member of a group of invertebrates called “protochordates.”

**FIGURE 7:**
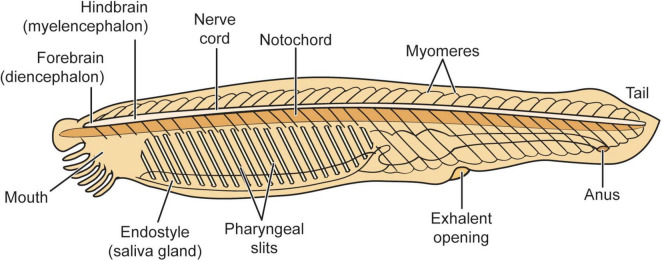
Amphioxus general neuroanatomy. Reproduced from Feinberg (2024), licensed under CC BY-NC-ND 4.0.

The adult brain of amphioxus, although much simpler than any vertebrate brain, could still have upward of 20,000 neurons. Numerous studies indicate that amphioxus is the closest living “proxy” for the ancestral chordate (animals with spinal cords) condition, and it has several brain structures that are likely progenitors or homologs of telencephalic, diencephalic, midbrain, and hindbrain structures that are found in later evolving vertebrates. Larval amphioxus also has an unpaired frontal eye in the midline that is the homologue of vertebrates’ paired eyes (Benito-Gutiérrez et al., 2021; Holland, 2015; Holland and Chen, 2001; Lacalli, 1996, 2004, 2008, 2010, 2021a, 2021b; Stokes, 1997; Stokes and Holland, 1995; Wicht and Lacalli, 2005; Candiani et al., 2012; Nicol and Meinertzhagen, 1991; Figure 7).

Evolutionary biologist Lacalli has done extensive studies on the amphioxus nervous system. The body surface of amphioxus is supplied with an assortment of epithelial sensory cells. It has a frontal eye that is probably too small and simple to be “image-forming.” Yet despite the relative simplicity of its nervous system when compared to ES3 animals, it is clear that amphioxus is capable of relatively complex adaptive responses that rely upon relatively simple neurohierarchically integrated sensing capabilities and responses (Pergner and Kozmik, 2017; Lacalli, 2018a,2018b).

For example, Lacalli describes the escape behavior in young amphioxus that provides an excellent model for the evolution of more complex approach-avoidance behaviors when compared to those of single-celled ES1 organisms. The animal’s brain features a critical integrative brain zone that is known as the *post-infundibular neuropile* that receives multiple inputs from the frontal eye as well as an assortment of other types of sensory neurons and other structures. The animal’s escape behaviors are induced most easily by mechanical stimulation of the rostrum, and sensory fibers originating there converge on the *primary synaptic zone*; along with input from the frontal eye, this regulates the animal’s escape response. However, while the complexity of amphioxus behavior falls well short of ES3 sentient animals, these sensorimotor escape behaviors are relatively more complex when compared to those of the single-celled organisms like *E. coli* described above.

### 5.2.2 Are ES2 animals sensing or sentient?

Although amphioxus has photoreceptors, touch receptors, chemoreceptors, and possibly some olfaction, it lacks image-forming eyes and hearing, so the isomorphic maps that are required to create *isomorphic mental images* are absent. Further, while there are early evolving and relatively simple midbrain, dien-mesencephalon, and even some telencephalon structures in amphioxus (Benito-Gutiérrez et al., 2021), these fall far short in complexity (see below) when compared to sentient ES3 animals. Finally, and importantly, in contrast to ES3 sentient animals, ES2 animals lack the affective infrastructure that is required for *sentient interoceptive-affective* feelings. Thus, based upon these considerations, I believe that amphioxus and other representative ES2 animals fall somewhere between ES1 and ES3 organisms, an emergent stage that I refer to as *presentient* (Feinberg, 2024).

### 5.3 Emergent Stage 3 (ES3): the emergence of sentient animals

**FIGURE 8:**
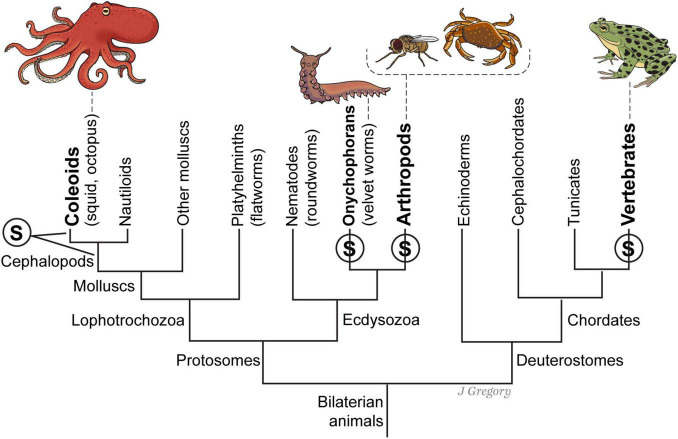
A simplified phylogenetic tree of the emergence of sentience in four different lines of sentient animals. At left, the two leaders extending from the S mean it could not be determined whether sentience evolved in the first cephalopod mollusks or else in the coleoid ancestor of squid, octopus, and cuttlefish. A similar uncertainty applies to the division between onychophorans and arthropods. Reproduced from Feinberg (2024), licensed under CC BY-NC-ND 4.0.

The evolutionary progression from sensing to sentience makes a significant leap between ES2 and ES3 along several evolutionary lines. This progression is the result of a remarkable increase of the neurobiological features that enable the emergence of sentience. Animals at this level include all vertebrates, coleoids (octopus), all arthropods including insects and decapods (such as crabs), and onychophorans (velvet worms) (Figure 8). Therefore, when we trace the evolution of sentience in ES3 animals, we see that neurobiological and hierarchical complexity is an obvious feature of their brains (Table 3).

**TABLE 3:** Emergent stage 3.

• Brain with increased number of neurons (approximately > 100,000)
• Many differentiated neuronal subtypes
• Expansion of neural hierarchies with extensive neural interactions
• Elaborated sensory organs with image-forming eyes, receptor organs for touch, hearing, smell etc.
• Centralized topographical maps create exteroceptive “sensory images”
• More developed neural infrastructure for affect and pain
• Centralized positive and negative valenced (sentient) affects including pain beyond simple nociception
• Increasingly non-reflexive (volitional) actions and globally directed behaviors

Animals with more neurobiologically evolved brains and sentience. Reproduced from Feinberg (2024), licensed under CC BY-NC-ND 4.0. First appearance 560-520 million years ago. Animals at this level: all vertebrates, coleoids (octopus), all arthropods including insects and decapods (such as crabs), and onychophorans (velvet worms). Novel neural structures and processes supporting the emergence of sentience.

#### 5.3.1 Neurobiological complexity and the emergence sentience

In general, when we compare ES3 animals to ES1 organisms and ES2 animals we witness a substantial increase in a variety of neurobiological features that are critical for the emergence of sentience. These include a marked increase in the *number* of the overall neurons in the nervous system, the degree to which these neurons display *specialized functions*, the number of *hierarchical levels* in the nervous system, and the degree of the *interaction* among these levels that is required for the emergence of a novel system feature like sentience (Table 4).

**TABLE 4:** Summary of the neurobiological variables that mark the progression from sensing to sentience.

• Increasing *number of neurons*
• Increasing degree of *specialized neural functions*
• Increasing *number of hierarchical levels*
• Increasing degree of the *interaction* between neurohierarchical levels

Reproduced from Feinberg (2024), licensed under CC BY-NC-ND 4.0.

I propose that these four critical variables can serve as a useful way to judge the *neurobiological complexity* of a nervous system that is characteristic of ES3 sentient animals and hence required for the emergence of sentience. This view is also consistent with numerous other opinions regarding the relationship between complexity and emergence. For instance, Vintiadis (2013) has emphasized that a system’s novel emergent properties are intimately linked to its degree of complexity. In addition, Ladyman and Wiesner discussed in their book on complexity science that “There is no conception of complexity or complex systems that does not involve emergence” (Ladyman and Wiesner, 2020, p. 73).

Further support for an intimate relationship between complexity and emergence comes from Mitchell, another expert on the subject of complexity, who goes so far as to suggest that a potential definition of a *complex system* would include the presence of emergent features in self-organizing systems such as living organisms (Mitchell, 2009).

All of the factors cited in Table 4 will inevitably increase the *neurobiological complexity* of a given nervous system. It also follows that these four factors create a significant increase in the likelihood of the *emergence of novel system features* (as enumerated in Tables 1, 2), one of which is sentience.

#### 5.3.2 ES3 animals and the punctuated emergence of sentience

Based upon the neurobiological and evolutionary features I have reviewed above, the evidence strongly supports the view that all ES3 animals (Figure 8) are sentient because they all possess the complex neurohierarchical features (Table 4) that enable the emergence of sentience (Table 5).

**TABLE 5:** A summary of the properties of sentience that are consistent with it as an emergent property of neurobiologically complex brains.

• Both sentience and emergent properties in general are *processes* created by the dynamic interaction of a system’s parts
• Sentience is an *aggregate system feature*, like emergent properties in general, that is not present in the parts of the system in isolation. Thus, emergent sentient properties are *novel* in comparison to the properties of the parts that create them
• Despite its aggregate origins, like emergence in general, the *substrate* of the parts of the brains of sentient animals are determinative factors that account for the differences in sentient “feelings”
• Hierarchical organization in general is a universal feature of all living things. Likewise, for sentience to emerge, there must be an exponential evolutionary increase in *neurohierarchical organization* that is characterized by innumerable specialized and interconnected parts and hierarchical levels

**FIGURE 9:**
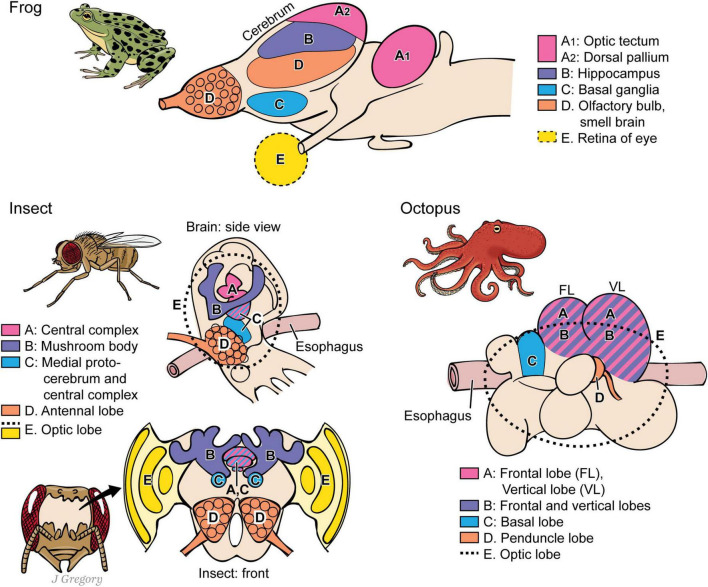
A comparison of the brains of three different lines of some proposed sentient animals. Pictured are the brains of a frog (vertebrates), an insect (arthropods), and an octopus (coleoids). Regions with similar functions for sentience are marked similarly in the three kinds of brains. Despite the similarities, the three brains evolved independently of one another. (A) image-based sentience; (B) memory; (C.) premotor center; (D) smell processing; (E) visual processing (illustration used with permission of ©Mount Sinai Health System).

First, there are important *common* neuroanatomical features possessed by all sentient ES3 animals. For instance, the common features among vertebrates, arthropods, onychophorans and cephalopods include: image-forming eyes and the capacity to form isomorphic mental images for *exteroceptive sentience* (Figure 1) and the neurobiological infrastructure for the creation of *interoceptive-affective sentience* for positive and negative emotional experiences, such as valence neurons and circuits (Figures 8, 9). Also, these clades have large brain regions for memory storage—with recalling memories being a part of conscious learning and spatial navigation (Mallatt and Feinberg, 2021).

There are additional common and important features that are shared by these clades of sentient animals. For instance, both NBE and Integrated Information Theory (IIT) posit that reciprocal or “circular” interactions between neural levels that are made possible by complex multi-level hierarchical nervous systems are key factors for the emergence of sentience. This point, centering around the reciprocal interactions, is also central to several other theories of consciousness (Baars et al., 2013; Ellia and Chis-Ciure, 2022; Lamme, 2006; Min, 2010).

Thus, despite the novelty of sentience, it is nonetheless remarkable that all of these common features at the ES3 stage are completely consistent with the features of emergence in biological and neurobiological systems in general. Thus, for sentience to emerge, the system’s parts must be physically united, integrated or at a minimum interacting in some fashion (Table 1) as well as possess *neurohierarchical systems organization* (Table 2) that are critical for this striking increase in the *novel emergent system properties.* This clear correspondence between the degree of emergence and the degree of neurobiological complexity supports the NBE hypothesis that sentience is a biologically emergent aggregate system feature that is created by later evolving and more complex nervous systems

However, additional support for the role of emergence in the creation of sentience also comes from the *diversity* across these clades; and while ES3 animal have neuroanatomical similarities, they also have anatomical differences among them (Figure 9). But note that an important property of all complex emergent systems is that more than one physical pathway can cause the same emergent end-phenomenon. This property is called *multiple realizability* or *multiple realization* (Bickle, 2020; Shapiro, 2004; Mallatt and Feinberg, 2021; Polger and Shapiro, 2016). This could help explain how consciousness evolved by convergent evolution in each of the distantly related taxa, vertebrates, arthropods, and cephalopods, yet still allow for some neuroanatomical diversity. Finally, there is the question of why this emergence occurred in a punctuated fashion? I chose these three stages because they are documented in the fossil record, and they also reflect the levels of emergent complexity of living organisms: 1. from non-life to prokaryotes; 2. one-celled to multicellular animals that then evolved nervous systems and simple brains; and 3. first animals with complex brains that I deem sentient.

There are several proposed reasons when and why this series of evolutionary steps occurred. ES1 was spurred when the first pre-cells gained the ability to reproduce and evolve via natural selection that natural selection that is a rapid driver of change. Stage 2 is hypothesized to have occurred when the Earth’s atmosphere and sea floor accumulated high enough levels of oxygen to support active, macroscopic animals. And Stage 3 is thought to have been spurred by the rise of predation and the “Cambrian arms race” (Ding et al., 2019; Erwin and Valentine, 2013; Feinberg and Mallatt, 2016; Fox, 2016).

Thus, there is good support and a scientific rationale for the proposed punctuated emergence of sentience from life in roughly three stages. But note these transitions are very broad in terms of evolutionary time and spanned billions of year of evolution.

## 6 Bridging the “explanatory gaps”

### 6.1 The first gap: NBE, the personal nature of sentience, and “knowin*g* what it’s like”

I have argued that NBE, as a biological-neurobiological-evolutionary theory of the natural emergence of sentience, can bridge the explanatory gaps between the *objective neurobiological* properties that create sentience and its *personal subjective features.* Here I discuss my approach to what I believe are the two major explanatory gaps—the *personal nature of sentience* and the *character of experience*—and offer a biological-neurobiological-evolutionary reconciliation for these philosophical problems.

#### 6.1.1 What Mary didn’t know

Let’s consider a well-known thought experiment that was proposed by philosopher Frank Jackson in the articles entitled *Epiphenomenal Qualia* (1982) and *What Mary Didn’t Know* (1986) that addressed the problem of the personal nature of sentience. Over the years, the philosophical questions that Jackson raised came to be known as the “knowledge argument” (Conee, 1994; Feinberg and Mallatt, 2020; Hasan and Fumerton, 2019; Jackson, 1982, 1986; Metzinger, 2003; Nida-Rümelin and O Conaill, 2019; Tye, 2002).

Jackson’s theoretical scenario is to imagine that there is a brilliant neuroscientist named Mary who knows everything that there is to know about the neurobiological physical and scientific properties behind color vision. However, Mary has been raised her whole life in a black-and-white room and thus never *personally experienced* color. The philosophical question is, when Mary is finally released from her room, and she *sees* color for the first time, what are the implications of what Mary “learns”?

**FIGURE 10:**
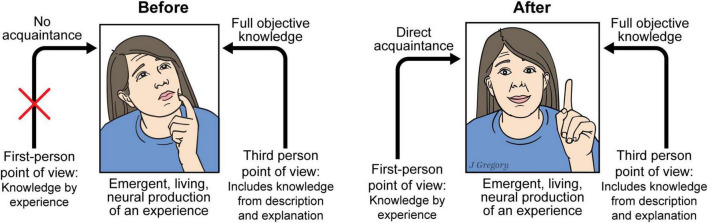
Mary, the emergence of sentience, and the explanatory gaps. Some kinds of knowledge can only be obtained by experience. In this context “Knowing” is of two types: experienced via *personal acquaintance* or by *objective descriptive or explanation*. Before Mary has direct experience of color (left) although she has full objective knowledge of brain processes, she lacks the first-person experience that she gains after she experiences color (right). The difference between “before” and “after” can simply be explained by the fact that only by *being* a living neural brain that produces the emergent process of experience can knowledge by acquaintance be acquired. Reproduced from Feinberg (2024), licensed under CC BY-NC-ND 4.0.

The particular type of “knowledge” about color that Mary initially possessed in her colorless room has been referred to as *knowledge by description*, a term that was coined by Bertrand Russell over a hundred years ago (Russell, 1910–1911; Russell, 1912; Russell, 1914). And this type of knowledge stands in direct contrast to what Russell referred to as *knowledge by acquaintance*, a type of knowledge that requires direct *personal experience*. It is *knowledge by acquaintance* that Mary gained upon her release from the colorless room (Figure 10).

In actuality, the problem is essentially the same one that was raised by Broad’s archangel. Both Broad’s archangel and Mary knew all the *facts* about the brain, yet the argument goes she would still be unable to *deduce* from those facts what color actually *feels* like. Hence, this is one gap between the objective facts of the brain and their relationship to direct sentient experience, e.g., the *personal nature of sentience*.

There are many interpretations and debates about Mary tells us about sentience or consciousness. These opinions are many and diverse (for instance, see Ludlow et al., 2004). While Jackson’s actual conclusions about Mary are subject to debate, one commonly raised question is whether the Mary problem is an argument against *physicalism*; that is, if personal sentient experiences cannot be *equated* with material brain states, then this creates a philosophical dilemma that for some leads to an argument against the “material,” “physical” basis of consciousness or mental functions.

Jackson initially posited that the implications of the Mary thought experiment indeed led to the view that a dualistic gap between the “mental” and the “physical” aspects of consciousness appeared scientifically irreconcilable.

However, subsequently, Jackson reversed this initial position and came to a radically different conclusion to the effect that the Mary experiment does not support an argument against *physicalism.* Of particular importance for NBE is *why* he changed his mind by arguing that an *aggregation* of physical elements could in fact be capable of creating phenomenal consciousness (Jackson, 2004, p. xvi). Note that this latter position is one of the central claims of NBE. This natural neurobiological emergence of sentience is represented in the center figures of Mary (Figure 10). Next, I explain how neurobiological emergence explains the first gap, the *personal nature of sentience.*

### 6.1.2 The evolutionary and biological emergent origins of the personal nature of sentience

While I agree that the personal nature of sentience appears to be a “hard problem,” I believe that when sentience is viewed as a *biologically emergent process*, its solution is surprisingly straightforward. First, as I have outlined in the timeline shown in Figure 2, the biological and evolutionary “seeds” of the *personal nature* gap date back to the emergence of the *personal life of an organism* that arose 3.5–3.4 billion years ago. It is here that we see the emergence of sensing as an emergent feature of all living organisms; and therefore—just like life—it is a personal emergent feature of the living organism. So, we can conclude that from the evolutionary pathway from life to sensing ES1 organisms to sentient ES3 animals, it is inevitable that once sentience emerges, it will be a *personal emergent system feature of the embodied individual organism*. Hence, sentience will have a naturally evolving and philosophically unproblematic personal nature.

In conclusion, regarding this first gap, from the biological, neurobiological and evolutionary standpoint, once we appreciate that sentience is inevitably a *personal emergent system feature of certain complex and evolved brains*, it follows that sentience is necessarily personal to the individual animal that creates it, as it is with ourselves.

### 6.2 The second gap: NBE, the *character of experience*, and why sentient experiences “feel” the particular way that they do

This second explanatory gap is explaining, with our knowledge of neuroscience, why “feelings” are experienced the *particular way* that they are. As Levine, put it: “There appears to be nothing about C-fiber firing which makes it naturally “fit” the phenomenal properties of pain, any more than it would fit some other set of phenomenal properties…. One might say, it makes the way pain feels into merely brute fact” (Levine, 1983, p. 357). Similarly, Chalmers (1996, p.5) wondered why, when listening to the sound of middle C played on a piano, the neural pathways from the ear to the auditory cortex are accompanied by any experience at all? Or experienced by a sound with tone and timber? (1996, p. 5). Why does the sound have the particular “character” that it does? First, it is clear, and no neuroscientist disputes, that the feeling of, for instance, the color red versus the pain of a pin prick is the result of the specific neurobiological functions of an individual’s nervous system. And I have argued that sentience (“feeling”) is the result of *emergent neural functions*. The different feelings that emerge from these brain processes are numerous and enormously complex, but to my mind there is no evidence that they are not entirely neurobiologically natural and specific to their *underlying neurobiological substrates.*

And as it is also clear that the neural pathways of color processing, sound processing, pain processing, affect and so on show enormous neurobiological differences; these diverse sensations should not—*and indeed could not—*have all have the same subjective “feels.” Why do neural states “feel” the way they do? I offer two primary explanations. The first part, as explained before, is that all sentience is a *unique personal emergent process of the individual organism;* so ultimately objective explanations can never be *equated with* subjective experiences. Second, asking “why” any neural state should be associated with a particular “feel” ignores the fact that various diverse “feelings” critically correspond to their diverse underlying neurobiological substrates.

Thus, the answer to the “character of experience” enigma, when viewed through the lens of NBE, is also surprisingly easy to understand; answering why red feels “red” versus the sound of a trumpet or a painful pinprick is clear: these feelings are both personally emergent and are *also* associated with different neurobiological substrates. And as is the case with all emergent system features, *the substrate and the functions of the parts determines their aggregate emergent features.*

### 6.3 The “experiential gap”

I conclude, based upon this line of reasoning, that there are not any real scientific “explanatory gaps” as described by Broad, Levine and Chalmers, and that the “Mary problem” is not actually describing a real scientific explanatory gap but rather represents an *experiential gap* between objective descriptions of nervous systems and the *emergent and biologically personal system features of sentience* in sufficiently evolved complex brains (Feinberg and Mallatt, 2020; Feinberg, 2024).

Thus, when the biological, neurobiological and evolutionary issues are properly understood and taken into account, the apparent “gap” between the *objective* brain and *subjective* experience is neither a scientific nor philosophical impasse. But at the same time, attempting to fully “objectify” the subjective experiential aspects of sentience is scientifically and philosophically unnecessary. In my view, the *experiential gap* is not in fact a scientific gap in our knowledge about how brains operate or create experience; rather, the *experiential gap* is the naturally occurring result of the biological, neurobiological and evolutionary emergence of sentience (Figure 2).

In summary, I propose that *Neurobiological emergentism* (NBE) can scientifically reconcile the conundrums that confront us when attempting to explain the relationships among the biological, neurobiological and evolutionary complexities of sentience with the two most commonly recognized “explanatory gaps.” And further, that resolving the controversies and perplexities surrounding the *explanatory gaps* with the emergence of an *experiential gap* is scientifically and philosophically unproblematic.

## 7 Neurobiological emergentism: comparison with some related theories

There are other theories of sentience and consciousness that relate to NBE. While these theories are primarily directed at explaining “consciousness” in general and are not specifically aimed at explaining the “feeling” aspects of sentience, two theories in particular relate to NBE and the emergence of sentience.

### 7.1 Biopsychism and the “Emergentist’s Dilemma”

The term *biopsychism* is generally credited to Haekel in 1892 (Haeckel, 1892; Thompson, 2022) and advocates the view that all living organisms are sentient. One recent version of this theory has been provided by Reber and colleagues. Reber called his theory the *Cellular Basis of Consciousness (CBC)* that holds that all living things down the single-celled organism at the ES1 level are sentient (Reber, 2019; Reber et al., 2023).

With reference to NBE, one of the central arguments for CBC is that consciousness could not have *naturally emerged* on an evolutionary pathway somewhere between a single-celled organisms like bacteria and sentient animals. From this, the CBC argument is that sentience must have been present in bacteria in the first place. Reber (2019) refers to this argument as the *Emergentist Dilemma*:

In sharp contrast to CBC, NBE asserts that single-celled organisms are not in fact sentient, and that while single-celled organisms are sensing, this is an early evolving emergent biological feature that can occur without any nervous system, much less sentience. Psychologist and neuroscientist Woodruff (2016) explained why this view is not supported by the evidence, and as I have argued earlier, the sensing capacities and the adaptive behaviors that are driven by these sensing capacities, such as those of *E. coli* described above (sections 5.1 and 5.1.1) can be fully explained by molecular chemotaxis mechanisms, and therefore these behaviors do not require nor support the presence of sentience in single-celled organisms.

In agreement with Woodruff’s view, I argue that in fact we can clearly and unambiguously trace an evolutionary progression from sensing to sentience (Figure 2), and that there is in fact no “*emergentist’s dilemma.*” Rather, NBE provides an *emergentist’s solution* to how and when the emergence of sensing occurs, and that all of the sensing behaviors of ES1 organisms ES2 animals as well as the sentience of ES3 animals are consistent and can be explained by the general principles of neurobiological emergence (Table 2).

### 7.2 NBE and integrated information theory

Integrated information theory (IIT) is a theory of consciousness originally proposed by Tononi (2004). The theory of IIT is complex and posits a number of axioms as well as numerous mathematical formulations (for some discussions see Merker et al., 2022; Oizumi et al., 2014; Tononi, 2015; Tononi and Koch, 2015; Tononi et al., 2016).

One of the central postulates of IIT is what Tononi refers to as “phi,” or symbolically designated as “Φ.” According to IIT, phi is a measure of “integrated information.”

#### 7.2.1 Common features between NBE and IIT

Despite the many fundamental differences between NBE and IIT, there are some interesting common features. For instance, one of the most striking aspects of the concept of phi is how strongly it invokes *standard emergent principles* even if the term “emergence” is not specifically and largely employed. Tononi (2015; Tononi and Koch, 2015) proposed that for a system to have a phi value above 0 it must be “unified,” “irreducible to its parts,” and the “whole must have cause-effects power above and beyond its parts.” But all these features of phi are widely accepted as *basic features of biological and neurobiological emergence in general* (Tables 1, 2) as well as my proposed features of animals with sentience (Tables 3–5).

Another common feature between NBE and IIT is that both theories note the essential role of reciprocal (“circular”) interactions between the neural “parts” or “levels” and “consciousness,” or in the case of NBE, sentience. But note that this point is not unique to either theory and is widely proposed by many theories of consciousness (Baars et al., 2013; Ellia and Chis-Ciure, 2022; Feinberg and Mallatt, 2016; Lamme, 2006; Min, 2010) and therefore may apply to many different approaches to the neurobiological basis of sentience or consciousness.

#### 7.2.2 Differences between NBE and IIT

On the other hand, when compared to NBE, the IIT criteria for consciousness is extremely low. IIT proposes that while any system or its parts whose integrated information less than Φ*^Max^* cannot be conscious, according to some interpretations of the concept of phi, virtually anything with interacting parts that has a phi value above 0 is therefore “conscious.” So, by this line of reasoning, consciousness could be ascribed to simple, inanimate entities like thermostats or even further down to a single proton with its interacting quarks (Koch, 2019; Mallatt, 2021; Tononi, 2008). Indeed, IIT asserts that such an extremely low degree of emergence or complexity is required for the creation of consciousness that it has been considered by many critics from various fields as endorsing a variety of *panpsychism* (Doerig et al., 2021; Fallon, 2016; Mallatt, 2021; Michel et al., 2019).

In the biological realm, based upon this extremely low criterion for “consciousness,” it has been suggested that even a single protein molecule as well as all living organisms, including all single-celled (ES1) organisms that possess emergent properties like simple sensing capabilities, which have phi values above 0, are conscious (Koch, 2019). But I have provided the behavioral and neuroanatomical evidence why this view appears to be incorrect.

Therefore, in contrast to IIT, NBE holds that it is the *degree and complexity* of biological and specifically neurobiological emergence that determines the emergence of sentience, not just the mere presence of *any degree* of emergence or any degree of “integrated information.” The neuroanatomical and behavioral evidence across many species (Figures 8, 9) provides strong support for this view. Therefore, I think that when compared to the low levels of “integrated information” as proposed by IIT, the emergence of sentience requires a far higher level of neurobiologically evolved and complex brains.

It also follows from this that another significant difference, related to the first, is that while IIT proposes its central factor phi is a *continuously* increasing function of the degree of “integrated information,” and this function progressively increases along with the increasing complexity of the system in question, NBE asserts that sentience actually emerges in *punctuated stages* from non-sentient life and does not emerge until the appearance of ES3 animals approximately 520 mya (Figure 2).

### 7.3 Does substrate matter for the emergence of sentience? NBE versus IIT

Another major and important difference between IIT and NBE relates to the role that the *physical substrate and properties* of the parts of a system play in determining what systems can in fact be sentient, or in IIT terminology, “conscious.” So, this raises the question of whether the substrate of a system—either its parts or the whole—*constrain* what systems can be sentient. Thus, according to some perspectives on IIT, the physical substrate of a system is irrelevant to both its creation and the manner in which it is experienced. Here is how Ellia and Chis-Ciure expressed it:

IIT allows consciousness to be realized in multiple substrates, in fact the substrate *per se* is irrelevant, as long as it specifies the integrated information described by the mathematical formalism of the theory (Ellia and Chis-Ciure, 2022, p. 5).

In contrast to this view, NBE proposes that the *living biological substrate* and features of the *neural parts* of sentient animals—via the constraints imposed upon them mandated by their biological evolutionary history—do indeed matter for the emergence of sentience. Just because a system is a functional aggregate of its parts does not mean that the features of the parts are irrelevant for the features of the collective whole. Indeed, the opposite is true: *all emergent system features in biology reflect and depend upon the functional features of the parts and their interactions.*

This may seem like an obvious point, but it is one that is frequently overlooked. No biologist would claim that the functional emergent properties of strands of DNA are not in part dependent upon the physical properties of the nucleotides of which they are composed and their interactions. Or that the specific emergent properties of, for example, the role that the lungs play in respiration, are not dependent upon of the physical properties of alveoli cells of which they are in part composed. Indeed, emergent reasoning plays a critical role in many biological investigations and biological theory in general (Herring and Radick, 2019; Rothschild, 2006; Salthe, 1985).

So why should the emergence of sentience (or consciousness) be any different? I have tried to demonstrate that the fundamental principles behind the emergence of sentience are not different in kind from any other biological emergent system feature. So, in contrast to the postulates of IIT, I argue that the *substrate of living things is also likely to be an essential prerequisite for the emergence of sentience*.

Consider that relatively biologically less complex living organisms, such as single-celled organisms, are alive, but for the reasons I have enumerated, they are not in my view sentient. Thus, according to this account, being a living organism with phi value far more than 0 is insufficient for sentience. On the other end of the spectrum, computers with advanced AI capabilities are very complex with astronomically high phi values via IIT criteria—or for that matter complex via any criteria—but they are neither alive nor sentient. So, neither life nor complexity in general (for instance as measured by Φ*^Max^*) alone ensure sentience or “something it is like to be.” I argue that as far as we currently know *everything that is sentient is both alive and neurobiologically complex*. There is the strong possibility that the substrate that enables the emergence of sentience very well might specifically require the biological features that enable the emergence of life *in addition to* complex neurobiological features.

Finally, if indeed, someday in the future, artificially intelligent entities do become sentient—something that admittedly we cannot definitively predict—I personally would venture a guess that even if such a thing were possible, whatever “sentient-like” features (if any) non-living, non-biological entities might display, as a result of the differences in their underlying substrates, these will not be “experienced” the same way that animal sentience is personally experienced.

### 7.4 Summary of these related views

In summary, I argue that the arguments in support of *biopsychism* and the “emergentist dilemma” do not pose scientific barriers to the emergentist NBE point of view. And in the case of IIT, while many of its postulates actually support many of the emergentist positions of NBE, in comparison to NBE, it sets the bar far too low for the emergence of sentience. In addition, despite its implicit endorsement of some emergentist principles, IIT fails to take into account the critical role that the substrate of the parts of a system play in the emergence of sentience. By this account, IIT does not solve the many of biological, neurobiological, evolutionary, and philosophical issues that NBE can explain.

### 7.5 NBE and some testable predictions

There are several ways that the postulates of NBE could be tested utilizing some theories that propose various statistical measures of consciousness, such as IIT.

For instance, IIT has applied sophisticated multichannel electroencephalogram (EEG) analysis to measure its metric of consciousness called phi in humans (Koch et al., 2016; Kim et al., 2018). These same techniques could, at least in principle, measure this parameter in ES3 animals such as lizards, crabs, and octopuses (Mallatt, 2021). NBE predicts these animals and humans show similar EEG patterns for calculating phi.

As argued above, phi could also represent one measure of the emergence and complexity of a system in that phi is a measure of the “unification, irreducibility, and the causal power of the whole system over its parts” (Tononi, 2015; Tononi and Koch, 2015) and that all of these criteria are features of the degree of emergence of a system (section 7.2.1). Therefore, both NBE and IIT would predict that the degree of phi corresponds to the likelihood of a system’s—and its nervous system—having consciousness. This outcome would actually lend support for both theories.

However, NBE would predict an important and testable difference between the two theories. As also noted earlier (section 7.2.2) IIT proposes that phi is a *continuously increasing function* of the degree of “integrated information” of a system and its “consciousness,” while NBE predicts that phi would show dramatic and measurable but *punctuated stages of increase* from non-sentient life to sentient animals.

There are other strategies that could test the postulates of NBE. In two recent papers, Irwin (2024a; 2024b) developed a behavioral profile that assigns quantitative measure to three proposed markers of consciousness*—volition*, *interaction*, and *self-direction*—the presence of which he quantified according to the *frequency*, *variety*, and *dynamism* of each. He described a “3 modes × 3 metrics” matrix to quantify and compare the degree of consciousness across different clades of animals. This approach, along with others that are designed to objectively measure the degree of consciousness (Bayne et al., 2024; Birch et al., 2020; Dung and Newen, 2023; Kaufmann, 2021, 2024), could be applied to test the sentient species proposed by NBE.

Finally, in an interesting and novel approach to testing for consciousness in animals, Ehret and Romand (2022) compared measures of consciousness in humans with some other animal clades. They analyzed previously published human data on several generally accepted measures of awareness and hence consciousness, such as latencies of evoked potential peaks and the presence of electroencephalogram gamma-waves as neural correlates of exteroceptive sensory awareness that were recoded while human subjects were attending to and responding to external stimuli. Then the authors sought these measures of awareness in other animals including several species of mammals, and one species each of birds, fish, cephalopods, and insects.

The authors found that the neural electrophysiological markers of consciousness from the human data were also present in the mammals (rats, mice, macaque monkeys, and dolphins) and the bird (crows). The data on the electric fish *Gnathonemus*, cephalopods (cuttlefish) and insects (fruit fly *Drosophila*) showed some evidence for “possible awareness,” however the authors felt that in these animals the results were inconclusive and further studies would be required to determine the nature of the brain activity under investigation in these animals.

However, if such additional studies did uphold the initial possible results, that would be consistent with the notion of vertebrate-arthropod-cephalopod consciousness as advocated by NBE.

## 8. Conclusion: NBE and the evolutionary, neurobiological, and philosophical approaches to the “explanatory gaps”

The personal nature of consciousness—what Searle (1992) referred to as its *ontological subjectivity*—lies at the heart of the “explanatory gap.” In my view, the “explanatory” gap is actually two gaps: first, the *personal nature of consciousness*; and second, the *character of experience*. I propose that both can be explained by integrating the relevant perspectives of the evolutionary, neurobiological, and philosophical approaches when trying to explain the subjectivity of sentience and consciousness.

First, let us consider the *personal nature of consciousness*. It is inevitable that once sentience emerges from life, it will necessarily be a personal emergent system feature of the embodied individual organism. As Thompson suggested (section 5.1), embodied life gives subjectivity its *personal nature*. That fact alone will determine that sentience will have a naturally evolving and philosophically unproblematic subjectivity since it is an emergent system feature of the life and neurobiology of the organism. In other words, there are no *evolutionary or biological gaps* in the emergence of the personal nature of sentience.

So, what about the second explanatory gap, the *character of experience*? This is the question of why “feelings” are experienced the *particular way* that they are. First, it is an indisputable fact that specific and varied “feelings”—while they all are “sentient”—are created by specific and varying neuroanatomical substrates. But what all these experiences *do* have in common is that they are all *emergent system features of complex* brains; and the different emergent feelings that are associated with these neuroanatomical systems will inevitably generate different “feelings.”

Now, let us consider these two “explanatory gaps” in conjunction. First, if sentient experiences are unique embodied personal emergent processes of the individual organism, this explains their personal nature without any evolutionary or neurobiological “explanatory gaps.” Second, since these diverse conscious experiences also vary according to their associated neuroanatomical substrates, they will not nor could they be experienced in exactly the same way, thus creating the different “characters of experience.” So, there is no neurobiological gap here either. Thus, if there are no gaps in either the evolutionary history or neurobiological explanations of consciousness, is there even a “gap” at all?

In my view, there is an *experiential gap* (section 6.3). This is what Russell (section 6.1.1) referred to as the distinction between *knowledge by description* and *knowledge by acquaintance*, and what John Searle (section 3.1) meant when he referred to the “first-person ontology” of consciousness.

The experiential gap does not represent a gap in our scientific knowledge about how brains operate or create experience; rather, the *experiential gap* is the naturally occurring result of the biological, neurobiological, and evolutionary emergence of sentience. And when the evolutionary history and the emergent neurobiology of consciousness and sentience are properly understood and integrated—and that process is well underway—the “explanatory gaps” between the *objective* brain and the *subjective nature of experience* do not pose any scientific or philosophical impasse.

Finally, I propose that *Neurobiological emergentism* (NBE) can help to scientifically reconcile the conundrums that confront us when attempting to explain the personal and subjective nature of experience as well as its objective biological basis. And the controversies and perplexities surrounding the two *explanatory gaps* can be resolved by positing the emergence of an *experiential gap*; but the experiential gap—in contrast to the traditional explanatory gaps—is scientifically and philosophically unproblematic.

## Data Availability

The original contributions presented in this study are included in this article/supplementary material, further inquiries can be directed to the corresponding author.
